# Identification of a Splenic Marginal Zone Lymphoma Signature: Preliminary Findings With Diagnostic Potential

**DOI:** 10.3389/fonc.2020.00640

**Published:** 2020-05-08

**Authors:** Jacob E. Robinson, Timothy C. Greiner, Alyssa C. Bouska, Javeed Iqbal, Christine E. Cutucache

**Affiliations:** ^1^Department of Biology, University of Nebraska Omaha, Omaha, NE, United States; ^2^Department of Pathology and Microbiology, University of Nebraska Medical Center, Omaha, NE, United States

**Keywords:** SMZL, marginal zone lymphoma, signature, lymphoma, diagnostic

## Abstract

Splenic marginal zone lymphoma (SMZL) is a rare, indolent non-Hodgkin's lymphoma that affects 0. 13 per 100,000 persons annually. Overall survival of SMZL is estimated to reach 8–11 years in most cases, but up to 30% of SMZL cases develop aggressive presentations resulting in greatly diminished time of survival. SMZL presents with a very heterogeneous molecular profile, making diagnosis problematic, and accurate prognosis even less likely. The study herein has identified a potential diagnostic gene expression signature with highly specific predictive utility, coined the SMZL-specific Gene Expression Signature (SSGES). Additionally, five of the most impactful markers identified within the SSGES were selected for a five-protein panel, for further evaluation among control and SMZL patient samples. These markers included EME2, ERCC5, SETBP1, USP24, and ZBTB32. When compared with control spleen and other B-cell lymphoma subtypes, significantly higher expression was noticed in SMZL samples when stained for EME2 and USP24. Additionally, ERCC5, SETBP1, USP24, and ZBTB32 staining displayed indications of prognostic value for SMZL patients. Delineation of the SSGES offers a unique SMZL signature that could provide diagnostic utility for a malignancy that has historically been difficult to identify, and the five-marker protein panel provides additional support for such findings. These results should be further investigated and validated in subsequent molecular investigations of SMZL so it may be potentially incorporated into standard oncology practice for improving the understanding and outlook for SMZL patients.

## Introduction

Splenic marginal zone lymphoma (SMZL) comprises <2% of all lymphoid malignancies (1–5), yet remains uncurable and challenging to diagnose accurately. SMZL typically progresses indolently, with an overall survival of 8–11 years among most patients (6–8), but up to 30% of cases will advance more aggressively, with 5–10% of cases progressing to diffuse large B-cell lymphoma (DLBCL), both of which result in decreased overall survival (9–13). SMZL presents extremely heterogeneously and, as a result, a myriad of differential diagnostic assessments are utilized in an attempt to accurately identify the malignancy (13, 14). Due to inconsistencies among most evaluations, the definitive diagnosis of SMZL is typically only recognized upon review of spleen histology following a splenectomy (13); an operation that should be exercised as an absolute last resort due to the impending associated health risks (15, 16). What's more, even among cases properly diagnosed with SMZL, accurate genomic prognoses are not currently possible.

In addition to the rarity and symptomatic heterogeneity of SMZL, few molecular identifiers are consistently presented among cases. Chromosomal losses at 7q account for 25–40% of cases, and gains at 3q are seen in 10–20% of patients, and the most common genetic mutations are among NF-kB regulators (30–40%), mutations of NOTCH pathway components (20–40%), and in KLF2 (30–40%) (17–21). While these mutations provide mechanistic indications for understanding pathogenesis, they lack clear diagnostic significance due to their variation among cohorts.

These aforementioned restraints have resulted in limited availability of SMZL clinical data, and consequently, large-scale genomic analyses of SMZL has been difficult. Thus, there exists a need to properly evaluate the potential for a diagnostic panel across multiple sample cohorts. Gene expression profiling and the associated analysis of its data have provided great potential for deciphering diagnostic panels among malignancies (22–24). Many panels have been identified among malignancies, and it has been shown that these panels can potentially provide clinical utility—from diagnosis, to prognosis, to treatment design. Glas et al. (25) demonstrated the validity of a 78 probe microarray for prognostic evaluation among a subset of breast cancer patients and utilized a platform that provided test results to patients in as little as 5 days. Gene expression panels have also been utilized in an effort to differentially diagnose non-Hodgkin lymphomas in preserved samples (26). These efforts demonstrate the demand and promise for the development of gene expression panels for diseases and disease states that are difficult to assess clinically, like SMZL.

The identification of novel genetic targets also allows for the evaluation of their utility as potential treatment targets. Increasing the number of targeted treatment options would enable the reduction of splenectomy usage as the primary treatment option in SMZL patients, thus improving patient experience. Splenectomy can result in a variety of complications in patients involving susceptibility to infection, cardiovascular complications, thrombosis, and increased oncogenesis potential (27–29). As these complications have become more apparent, the decision to conduct splenectomy has begun to be reconsidered but without reliable diagnoses little can be changed (22). Hence, a gene expression panel that can be evaluated from a splenic biopsy rather than full splenectomy may point to other treatment alternatives that have shown success in SMZL patients, without the adverse splenectomy consequences (30).

Furthermore, genetic markers that can be utilized for diagnostic and prognostic use and can provide clear differentiation of SMZL cases against related malignancies and control samples, offers significant utility in an era of precision medicine (22–24). This study identified genetic markers and pathways that uniquely identify SMZL. Consequently, we report what we call the “SMZL Splenic Gene Expression Signature (SSGES)” as a gene expression panel with potential for use in SMZL diagnosis, and recommend EME2, ERCC5, ZBTB32, USP24, and SETBP1, for further investigation into their potential role in lymphomagenesis.

## Methods

### Gene Expression Data Collection and Collation

Gene expression datasets and profiles were extracted from the National Center for Biotechnology Information's Gene Expression Omnibus (GEO) database as well as from a collaborating laboratory. A description and record of all datasets obtained for this study can be found in (Supplementary Table 1). All datasets were downloaded as raw data (.CEL) and were uploaded concurrently into *BRB ArrayTools*. Additionally, samples were only selected for the gene expression classification if they were samples taken from a secondary lymphoid tissue site such as LN or Spleen, due to influence of the tumor microenvironment; consequently, any peripheral blood samples were removed. In total, 437 gene expression profiles from 13 different datasets were used for analysis in the present study, with 42 of those samples designated as SMZL.

### Data Preparation

Data were downloaded from GEO as individual datasets. In order to minimize batch effects, data were only selected if the *Affymetrix GeneChip Human Genome U133 Plus 2.0 Array* was used for the microarray platform. The. CEL files underwent MAS5.0 normalization, log2 transformation, and quantile normalization. No microarray spot filters were applied to the dataset, but the gene filter: exclude if <10% of expression data values have at least a 1.5-fold change in either direction from the gene's median value or if percent missing exceeds 60% at any gene, was applied. Replicate spots on the array were averaged and multiple probes/probe sets were reduced to one per gene symbol. This was selected by the maximally expressed probe as measured by average intensity across array, as previously described (31). Once collated, the data was labeled based on sample ID, tissue, and sample type (CSP-control spleen, DLBCL-Diffuse Large B-cell Lymphoma, EMZL-Extranodal Marginal Zone Lymphoma, FL-Follicular Lymphoma, MCL-Mantle Cell Lymphoma, NMZL-Nodal Marginal Zone Lymphoma, or SMZL). GEPs were then assigned CSP, SMZL, or tissue B-cell lymphoma (TBCL) for signature comparisons.

### Calculation and Selection of the SMZL-Specific Gene Expression Signature

Unsupervised clustering was conducted on all 437 samples using all available filtered genes. Sample type designations were assigned in order to differentiate each malignancy among this gene expression cohort. Genes were centered and scaled, and the samples were clustered by one minus correlation, based on average value, to determine linkage.

SMZL GEPs were then compared against CSP GEPs via univariate class comparison. No additional gene filters were applied, and the analysis was conducted at a significance level of 0.001. This univariate comparison was replicated between SMZL and TBCL samples.

All probes highly significantly, differentially expressed (*p* < 1e−7) in each respective class comparison were then selected for and cross compared to identify differentially expressed genes present in both datasets. Significance analysis of microarray (SAM) was then conducted on the most differentially expressed gene probes, as was recommended (32, 33). SAM was run using a target proportion of false discoveries at 0.01 and by completing 100 permutations. SMZL was first compared to CSP using the 164 genes highly differentially expressed, and the process was then repeated between SMZL and TBCL. These lists were cross-compared one final time, and 135 genes were identified to be uniquely, differentially expressed in SMZL against both, CSP and TBCL comparisons and identified as the SMZL Splenic Gene Expression Signature (SSGES) (Supplementary Table 2).

Multiple Methods prediction analysis was utilized through the *BRB Array Tools* platform. The SSGES was the designated gene list for predictive comparison. The alpha value for the multiple methods analysis was set to 0.01, and all predictive models were utilized and reported in the analysis. Functional pathway analysis was then conducted on both respective class comparisons. The functional and network analyses were generated using *Ingenuity Pathway Analysis* (34).

### Gene Expression Replication Cohort

In order to verify the predictive value of the SSGES, a replication gene expression cohort was utilized. All datasets were acquired from GEO, and all normalization, filtering, and design methodologies followed procedures of the initial test dataset. All sample information is provided in (Supplementary Table 3). Predictive analysis was also conducted as described above for the replication cohort.

### Protein Signature Extraction

For the development of a protein signature, the SSGES was condensed to the most impactful markers for evaluation in patient samples. This was completed using the total percent impact score (TPIS) in conjunction with *The Human Protein Atlas* in order to identify which markers would be optimal candidates for IHC (35).

This study proposes the calculation and implementation of a TPIS for ranking the differentially expressed markers. The TPIS for each differentially expressed gene was calculated by ranking each probe by its SAM score (achieved during the SAM analyses) through assigning the percent rank to each marker based on their score. For example, *IL7* presented the highest SAM score [*d*(*i*) = 7.852] when SMZL was compared with CSP, and so it was assigned an impact score of 1.0. *ZNF763* had the second highest SAM score [*d*(*i*) = 7.196] in the same comparison. In order to weigh each gene probe equally on its impact on total score, TPIS must be assigned based on SAM score instead of numerical order. Hence, ZNF763 had a TPIS of 0.91645, as that is equivalent to the percent rank of SAM score. This was done for all up-regulated gene probes as well as all down-regulated gene probes for SMZL vs. CSP and SMZL vs. TBCLs. The sum of these scores were then taken to identify the TPIS for both up- and down-regulated genes in the signature (Supplementary Table 2). In order to further evaluate the markers potential as protein assay candidates, the genes identified on the SSGES were then researched on *The Human Protein Atlas* for protein, spleen-specific expression in control tissues (35). Additionally, the chromosomal location of the genes was investigated and considered to evaluate if chromosomal aberrations could be responsible for the alteration in gene expression. Targets for iimmunohistochemistry (IHC) were identified by taking into account each of the aforementioned TPIS, chromosomal characteristics, and protein expression, as reported by *The Human Protein Atlas*. This resulted in identification of five key markers (ZBTB32, EME2, SETBP1, ERCC5, and USP24) that could be feasibly evaluated by IHC, based on resource and time availability.

### Immunohistochemistry Patient Samples

Tissue samples were provided by the Lymphoma Study Group at the University of Nebraska Medical Center (UNMC). Tissues were provided as either unstained slides or as paraffin embedded tissue blocks. There were 17 total samples provided, 6 SMZL, 6 control spleen, and 5 other B-cell lymphoma splenic samples [1 DLCL-C, 1 DLCL-NC, 1 FL, 2 follicular medium cell (FM)]. Full listing with available prognostic information can be found in (Supplementary Table 4). Each case was previously diagnosed by a physician then confirmed by pathologist upon removal via histology. Cases were also validated via IHC of immunophenotypic markers characteristic of SMZL (Supplementary Figure 1). This study had full approval for its completion through the institutional review board (IRB) at UNMC, and of the Fred and Pamela Buffett Cancer Center, and the Nebraska Lymphoma Study Group Tissue Bank #441-18-EP.

### Immunohistochemistry

IHC was conducted using the *Roche Diagnostics ULTRA Discovery* IHC platform housed at the UNMC Tissue Science Facility. Each slide contained 5 microns thick, paraffin embedded, sections of splenic tissue. The antibody and IHC information is provided in (Supplementary Table 5), inclusive of antibody, company of origin, catalog number, antigen retrieval time in minutes, optimized dilution, and antibody staining time in minutes. Each condition was optimized for staining 5-micron splenic sections. Optimization controls for EME2 and USP24 were conducted on kidney and liver, respectively. Hematoxylin counter-staining, dehydration, and re-application of cover slip was done using the Tissue-Tek Prisma platform.

### Scoring, Protein Comparison, and Prognostication

All slide scans were taken at 40X magnification before being analyzed using VENTANA Image Viewer. Slides were analyzed by three researchers independently. The slide order was randomized, and identifiers were blinded to eliminate researcher bias. Researchers were shown five separate screen-shots at 20X magnification for each separate slide and scored staining intensity from 1 (no staining) to 5 (diffuse, complete tissue staining) for each image. This scoring was then averaged between the three researchers for each image and staining intensity scores (SIS) were assigned to each image. The five images per sample were then averaged, and each sample was given an overall staining intensity score (OSIS). Each lymphoma diagnosis was then validated one final time by a trained pathologist prior to cohort comparison. Data comparisons between CSP, TBCL, and SMZL sample groups was done using Student's *t*-test.

## Results

### SSGES Delineation

Gene expression has been utilized as an effective technology for deciphering diagnostic and prognostic subgroups among B-cell lymphomas (23, 36). Despite previous efforts to evaluate the gene expression of SMZL cases (22, 37), definitive biomarkers have not been well-established. We analyzed 437 gene expression profiles, comparing control spleen (CSP), other secondary lymphoid organ B-cell lymphoma (TBCL), and SMZL samples. TBCL samples included diffuse large B-cell lymphoma (DLBCL, follicular lymphoma (FL), mantle cell lymphoma (MCL), nodal marginal zone lymphoma (NMZL), and extranodal marginal zone lymphoma (EMZL), for comparison.

**Figure 1:**
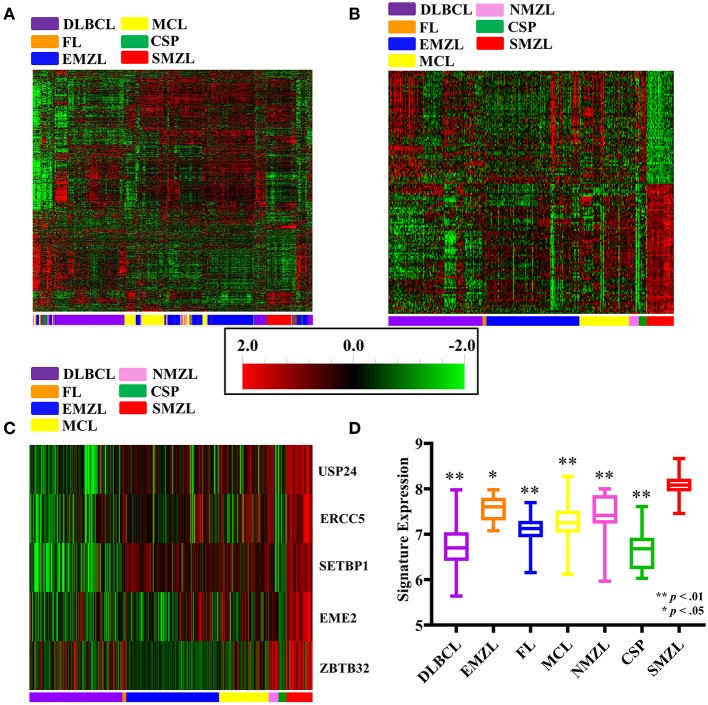
Gene expression dataset results shown. All heatmaps correspond to scale located in the center of the figure [2.0 (red) – −2.0 (green)]. All sample types are listed according to the color they correspond to located on the *x*-axis of each heat map: DLBCL (purple), MCL (yellow), FL (orange), CSP (green), EMZL (blue), SMZL (red), and NMZL (pink). **(A)** Unsupervised clustering of all gene expression samples assessed. **(B)** Supervised clustering by sample type for the 135 gene SSGES. **(C)** Supervised clustering of the five probe IHC panel. **(D)** Composite five probe IHC panel expression comparison.

In order to evaluate whether SMZL possessed a unique genomic profile, unsupervised clustering was conducted at a significance level of 0.001, on all 437 samples across the 16,272 available genes. SMZL cases clustered together when analyzed, suggesting there is a unique, delineating molecular signature by which SMZL can be more accurately diagnosed, if deciphered (Figure 1A).

In order to identify a unique SMZL genetic signature, univariate class comparisons were completed. The comparison between SMZL and CSP produced 5,707 gene probes that possessed a significant difference in expression, and SMZL GEPs compared to the TBCLs returned 6,477 gene probes. All probes highly significantly, differentially expressed (*p* < 1e−7) in each respective class comparison were then selected. The comparison between SMZL and CSP produced 1,056 highly differentially expressed genes, while the greater significance between SMZL and TBCL returned 2,374. These two lists then underwent cross-comparison to identify genes that were differentially expressed in SMZL from both datasets. This resulted in 164 genes that were differentially expressed in SMZL when compared with both CSP and TBCLs.

SAM was then conducted using the 164 gene signature to reduce the FDR by conducting gene-specific *t*-tests independently, as opposed to the class comparison *t*-test across the full dataset, further corroborating that the identified differentially expressed gene probes are not selected randomly. Using SAM, 147 and 148 genes were differentially expressed in SMZL when compared with CSP and TBCLs, respectively. These lists were cross-compared one final time, and 135 genes were identified to be uniquely, differentially expressed in SMZL against both, CSP and TBCL comparisons. This collection of 135 genes were coined the “SMZL specific Gene Expression Signature (SSGES)” (Supplementary Table 2, Figure 1B).

### Predictive and Functional Analysis

Multiple methods for predictive analysis were conducted to determine the fidelity of our diagnostic tool. This was evaluated for SMZL against CSP and TBCL separately to assess the predictive value of the 135 gene SSGES. For each method of predictive analysis to delineate SMZL from CSP was correctly classified 100% of the time. And, when this same SSGES signature is used to predict diagnosis between SMZL from other B-cell lymphomas (TBCL), it was correct 98% of the time (Supplementary Table 6).

### Gene Expression Replication Set: Ensuring Fidelity and Reliability of SSGES

A gene expression replication set containing 705 samples was organized independently of the original SSGES delineating cohort (Supplementary Table 3). The replication cohort contained secondary lymphoid organ B-cell lymphoma samples from datasets not used in the original analysis set as well as splenic controls and the original 43 SMZL samples. Due to the limited sample number of SMZL samples, all samples were used in the original and replication cohort, but new comparison samples were utilized. While this limits the ability to delineate new signatures, the replication cohort demonstrated the strong predictability of SMZL samples against other B-cell lymphoma samples and controls not originally used in signature delineation. Multiple methods predictive analysis was used, and with similar effectiveness to the original analysis, SMZL samples could on average be correctly differentiated from CSP and TBCL samples 98 and 95%, respectively (Supplementary Table 6).

### SSGES Protein Evaluation

To provide validation of the gene expression signature and enhance the impact of the study, five markers were selected to provide clinical utility as a protein assay for diagnostic purposes and to investigate for SMZL-specific treatment targets at the protein level. These markers were also evaluated alongside patient data to assess the prognostic significance of their increased expression. The five markers selected to be evaluated using IHC: *EME2, ZBTB32, ERCC5, USP24*, and *SETBP1*. Multiple methods predictive analysis was conducted using this five-gene cohort, with correct classification reaching ~95% in the original cohort, and ~90% when compared to the validation cohorts, indicating the potential for this much smaller panel to demonstrate diagnostic utility (Supplementary Table 6, Figure 1C). Lastly, these five markers demonstrated significantly higher expression in SMZL cases when compared against each malignant cohort individually, further demonstrating their selectivity for SMZL (Figure 1D).

**Figure 2:**
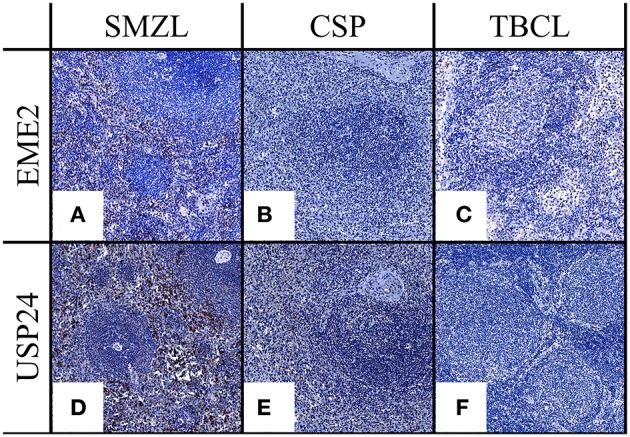
**(A–C)** EME2 IHC expression comparing SMZL, CSP, and TBCL. **(D–F)** USP24 IHC expression comparing SMZL, CSP, TBCL. All images at 20X.

**Figure 3:**
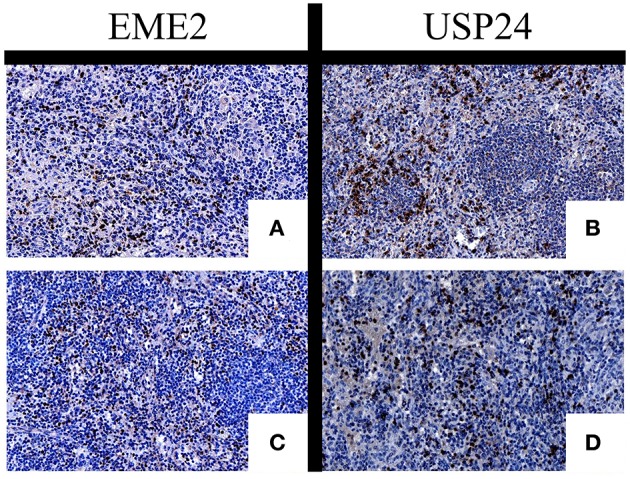
IHC of EME2 **(A,C)** and USP24 **(B,D)** demonstrating location of positive staining location within SMZL samples.

Following staining and respective scoring, expression of each marker was compared. EME2 was shown to have significantly higher expression in SMZL samples when compared with CSP (*p* = 0.05) or TBCL (*p* = 0.01) cohorts (Figures 2A–C), and USP24 demonstrated significantly higher expression in SMZL when compared to CSP (*p* = 0.02; Figures 2D,E). USP24 expression also appeared increased in SMZL compared to TBCL samples (*p* = 0.10) but was not statistically significant (Figures 2D,F). For both USP24 and EME2, increased expression was consistently observed within the interfollicular zones of SMZL cases (Figure 3), but marginal zone staining was only recognized sporadically.

**Figure 4:**
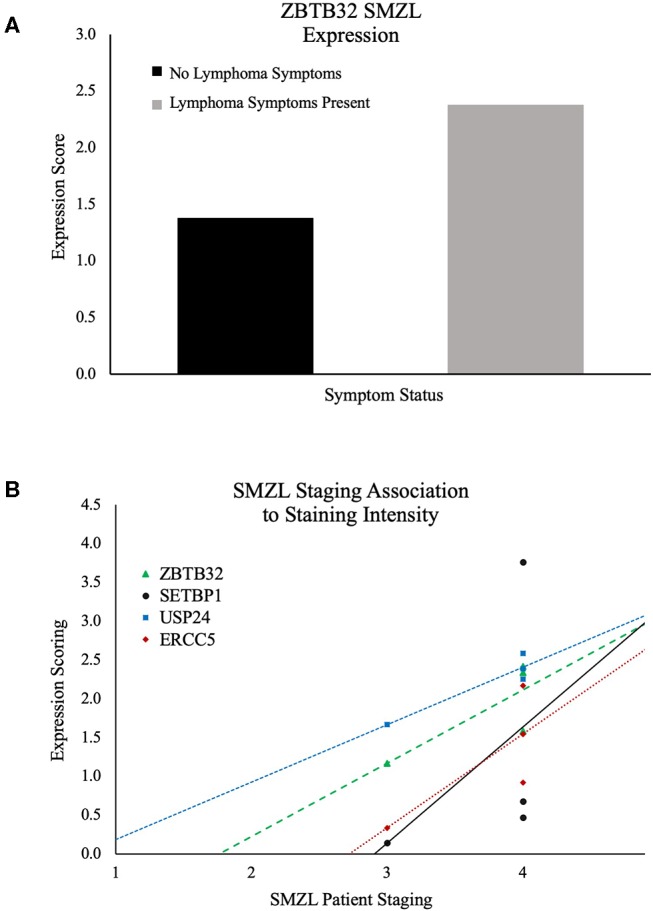
**(A)** ZBTB32 expression comparison among SMZL samples. Black: no lymphoma symptoms. Gray: lymphoma symptoms. Demonstrating increased ZBTB32 expression in SMZL cases that are concurrently presenting with lymphoma symptomology. **(B)** Association of patient staging and staining intensity for ZBTB32, SETBP1, USP24, and ERCC5 in SMZL cases.

The remaining markers did not present with statistically significant differential expression (Supplementary Figure 2), but ZBTB32, SETBP1, and ERCC5 were each indicative of possible prognostic value for SMZL cases. ZBTB32 showed significantly higher expression in SMZL cases with lymphoma symptoms present (*p* = 0.04; Figure 4A). Expression scoring values for ERCC5 (*r* = 0.76), SETBP1 (*r* = 0.44), and ZBTB32 (*r* = 0.78) in SMZL cases correlated with the patients' staging upon tissue extraction. (Figure 4B), and USP24 increased expression also correlated with increased staging of SMZL (*r* = 0.93) (Figure 4B). Further, SETBP1 expression also correlated with increased staging of the TBCL samples (*r* = 0.66), indicating its potential as B-cell lymphoma marker in multiple subtypes. Each of these markers' increased expression correspondence with severity of symptoms among the SMZL cases, points to their potential use as prognostic markers. Thus, they warrant further investigation among expanded patient cohorts to corroborate these preliminary findings.

## Discussion

In this study, a 135-gene signature coined the SSGES that demonstrates clinical potential for identification of SMZL was identified. Even more importantly, novel biomarkers have been identified via IHC that could provide diagnostic, as well as prognostic utility for SMZL patients. Given the considerable dearth of information for both diagnosis and prognosis of SMZL in the literature, it's expected that these findings will significantly contribute to patient outcome. Importantly, when we replicated the SSGES against new data sets and tested their predictability and fidelity, they yielded 90–100% accuracy of identifying SMZL, well-above current clinical markers and methods. These results provide preliminary findings that must be further validated in expanded investigations and should guide future inquiries into understanding SMZL pathogenesis.

Many treatment options have been proposed and are being further developed in marginal zone lymphomas, with significant improvement in SMZL relapse and progression free survival rates (38–41). Currently, many of these drug therapies will be tried in SMZL cases prior to resorting to a splenectomy as a treatment option, but there is still limited genetic information driving these decisions (20). In order to force a switch in treatment regimens and recommendations, a genetic panel must be produced that provides diagnostic and prognostic value for identifying SMZL. This study has identified such a panel, through meta-analysis of gene expression profiles among SMZL cases against other B-cell lymphomas and splenic controls. This panel must be further clinically validated and assessed for potential usage systematically, but the high predictive success of the panel against each comparison set is promising for the prospect of future implementation.

While each of the markers investigated in the present investigation are understudied, they each display mechanistic potential as drivers of SMZL carcinogenesis. EME2 in conjunction with MUS81 initiate replication fork restart (42), indicating its increased expression could be exploited by cancer cells for enhanced DNA replication. USP24 has been shown to play a role in anti-apoptotic tumoral activity in myeloma cells and displayed increased expression in cells displaying increased drug resistance (43). USP24 has also been shown to be a regulator of tumor microenvironment signaling and has been proposed as a potential therapeutic target due to this role (44). The mechanisms collectively demonstrate that its increased expression could play a role in SMZL pathogenesis. Increased ZBTB32 expression has been identified among memory B-cells (45), and thus, its increased expression among symptomatic patients may be due to an increased presence of the memory B-cell based SMZL cells, a commonly proposed cell-type of primary SMZL tumor cells. Polymorphisms at *ERCC5* and disrupted expression have been reported to increase solid tumor carcinogenesis due to the disruption of DNA excision pathways (46, 47), and in an IHC investigation study, *ERCC5* encoded XPG was shown to have positive expression in breast, ovarian, and sarcoma samples (48). Lastly, SETBP1 activation has been shown to play a role in leukemic progression and development. Missense mutations of *SETBP1* have been shown to increase the stability of the SETBP1 protein and result in its subsequent over expression in many myeloid neoplasms (49). Further, SETBP1 overexpression has been associated with shorter overall survival in leukemic patients (50). It is proposed that SETBP1 executes its oncogenic properties through suppression of tumor suppressor genes and by inducing increased myeloid cell development and proliferation (50, 51). Each of these mechanisms provide evidence that the demonstrated over expression of SETBP1 in this study may play a role in oncogenesis and should also be investigated further for its mechanistic value among SMZL patients. A more concise record of the potential biological impact that these markers provided is listed within Table 1.

**Table 1:** Proposed biological impact of markers selected for IHC panel.

**Gene**	**NCBI gene ID**	**Biological function (citation)**
*EME2*	197342	Replication fork restart (42)
*ERCC5*	2073	DNA excision disruption (46, 47)
*SETBP1*	26040	Cellular proliferation and development promotion (50), tumor suppressor suppression (51)
*USP24*	23358	Cell survival (43); Tumor microenvironment regulation (44)
*ZBTB32*	27033	Memory B-cell expression (45)

Upon evaluation of IHC, a significant amount of the respective protein expression occurred within the interfollicular zones of the spleens, rather than exclusively within the tumor cells, indicating the markers' potential role as tumor environment effectors. The importance of understanding the tumor environment has become increasingly relevant, as demonstrated by the pursuit of therapies that can be utilized to circumvent its impact on lymphoma progression (52). Despite the markers' inconsistent expression as identifiers of SMZL tumoral cells themselves, this increased interfollicular expression corroborates the increased gene expression observed in the splenic tissue SMZL samples. This increased expression of these proteins within the tumor environment should be considered for their possible role in lymphomagenesis.

This study identified markers that provide potential clinical significance in delineating SMZL from other B-cell lymphomas and improving the biological understanding of SMZL. Future studies much be conducted to validate and expand the information reported in this manuscript. The intention of this study was to identify markers that could assist in diagnosis of SMZL and provide empirical data for weighing treatment options and prognostic outlook. Thus, while this study identified potential markers that may play a role in SMZL pathogenesis and even progression, significantly more work must be completed to fulfill the goal of clinical utility. Mechanistic studies utilizing *in vitro* B-cell lymphoma models could assess the functional impact of increasing the expression of each of the protein markers discussed above. Additionally, evaluating the expression of these markers in expanded SMZL as well as other lymphoma cohorts not utilized here would further validate these results. Increased prognostic value would also come from evaluating survival time, treatment outcomes, and treatment reactivity along with SSGES expression.

Several limitations arose upon completion of the study. First, the authors were limited to the samples and respective sample information that was available for use within the *GEO* datasets. Ideally, the signature could be derived against additional other splenic derived lymphoma subtypes such as hairy cell leukemia variant, hairy cell leukemia, hepatosplenic T-cell lymphoma, macrophage-rich large B-cell lymphoma, and more DLBCL cases presented in the spleen (53, 54), but these data were unavailable at the time of study completion. Additionally, there was no available information regarding the status and source of control spleens used in this study for gene expression as well as protein expression evaluation. In order to necessitate a splenectomy there must be critical circumstances at hand, and thus, while the spleens did not present with another lymphoma, it is difficult to know the true molecular profile they present due to the variable circumstances that resulted in their availability. The authors were also limited by the number of samples utilized for the IHC investigations. Due to the rarity and indolent presentation of SMZL, sample availability is extremely limited. Hence, the prognostic and diagnostic implications of these results are disclosed as preliminary until further validation in additional sample cohorts. Lastly, the gene expression analysis used herein was limited to exclusive use of Affymetrix probes. Thus, additional gene expression and genomic sequencing data executed using other platforms were not considered for analysis. This limited the study sample size provided and the depth of information that could have been gleaned from a wider array of analysis platforms. Further investigations utilizing data across multiple platforms would provide validation to these results and would further enhance clinical utility of the disclosed SMZL signature. Each of these limitations were recognized throughout the completion of this study. In an attempt to combat them, multiple levels of replication were utilized, samples originating from numerous study sites and regions were included, highly significant statistical filtering was conducted, and the SSGES was analyzed against an independent validation gene expression cohort for its accuracy. These efforts and subsequent results suggest that the SSGES is a reliable tool for diagnosis of SMZL when compared against other marginal zone lymphomas and control spleen samples.

In conclusion, this study has identified a diagnostic gene expression signature that presents many potential markers for diagnosis of SMZL patients from a splenic biopsy. It has also identified five novel protein markers, EME2, ERCC5, SETBP1, USP24, and ZBTB32, that appear to play a role in SMZL pathogenesis. Each of these results warrant further investigation for their broader roles in B-cell lymphoma biology.

## Data Availability Statement

The datasets generated for this study can be found in the NCBI Gene Expression Omnibus (GSE12195, GSE16024, GSE2109, GSE23501, GSE35348, GSE35426, GSE53820, GSE55267, GSE57520, GSE7307, GSE93291, GSE146814).

## Author Contributions

Conceived and designed the experiments: JR, JI, and CC. Performed the experiments: JR and AB. Analyzed the data: JR and CC. Reviewed and validated pathology: TG. Wrote the paper: JR and CC. Reviewed the manuscript: JR, TG, AB, JI, and CC.

## Conflict of Interest

The authors declare that the research was conducted in the absence of any commercial or financial relationships that could be construed as a potential conflict of interest. The handling editor declared a past co-authorship with several of the authors, JI and AB.
