# Supplementary material for: Identification of a Splenic Marginal Zone Lymphoma Signature: Preliminary Findings With Diagnostic Potential
Source: Front Oncol. 2020 May 8;10:640. doi: 10.3389/fonc.2020.00640 (PMC7225304; doi:10.3389/fonc.2020.00640)
Supplement: Supplementary file 1 [file Table_1.docx]

**Supplementary Table 1. Gene Expression Datasets.**

All datasets utilized in this study are listed. The reference dataset ID for GEO, the amount and subtype of samples included, and the PubMed ID for publications associated with the data are provided.

| **GEO Dataset ID** | **Sample Type (n)** | **Associated Reference PMID** |
| --- | --- | --- |
| GSE12195 | DLBCL (73) | 19412164, 19965633, 21156281, 21390126, 22137796, 28314854 |
| GSE16024 | FL (7), MCL (7) | N/A |
| GSE2109 | Control Spleen (4) | N/A |
| GSE23501 | DLBCL (69) | 20610814 |
| GSE35348 | SMZL (27) | N/A |
| GSE35426 | SMZL (14), FL (5), MCL (5) | 23028731 |
| GSE53820 | FL (79), DLBCL (2) | 24357726 |
| GSE55267 | FL (51) | 24634383 |
| GSE57520 | Control Spleen (3) | 25057852 |
| GSE7307 | Control Spleen (5) | N/A |
| GSE93291 | MCL (20) | 28291392 |
| GSE146814 | MCL (44) | 22490335 |
| GSE146814 | SMZL (1), NMZL (15), EMZL (6) | N/A |
|  | Total: 437 |  |
