# Supplementary material for: Identification of a Splenic Marginal Zone Lymphoma Signature: Preliminary Findings With Diagnostic Potential
Source: Front Oncol. 2020 May 8;10:640. doi: 10.3389/fonc.2020.00640 (PMC7225304; doi:10.3389/fonc.2020.00640)
Supplement: Supplementary file 2 [file Table_2.docx]

**Supplementary Table 2. SSGES gene listing.**

The table is separated into two sub-tables (A. & B.). A. All genes that are significantly over-expressed in SMZL compared with CSP and TBCL. B. All genes that are under-expressed in SMZL compared with CSP and TBCL. Both tables list the total percent impact score (TPIS) for each gene, the SAM score of the gene expression in SMZL compared to CSP and TBCL [d(i)], and the fold-change of the gene expression in SMZL against CSP and TBCL. Bold designates gene targets selected for IHC validation.

**Supplementary Table 2A. SSGES genes uniquely over-expressed in SMZL.**

| **Gene** | **TPIS** | **CSP d(i)** | **vCSP Fold-change** | **TBCL d(i)** | **vTBCL Fold-change** |
| --- | --- | --- | --- | --- | --- |
| *IL7* | 1.786 | 7.852 | 6.820 | 3.182 | 2.990 |
| *ZNF763* | 1.727 | 7.196 | 2.500 | 3.283 | 2.190 |
| ***ZBTB32*** | 1.696 | 5.463 | 4.400 | 4.050 | 3.180 |
| *CXorf21* | 1.595 | 4.899 | 2.840 | 3.931 | 2.660 |
| *MINOS1P1* | 1.542 | 5.000 | 2.560 | 3.667 | 2.690 |
| *RABEP2* | 1.535 | 5.433 | 2.530 | 3.415 | 2.540 |
| ***SETBP1*** | 1.533 | 6.719 | 3.370 | 2.745 | 2.290 |
| *SYK* | 1.513 | 6.112 | 3.200 | 2.976 | 2.150 |
| *CARF* | 1.486 | 5.451 | 2.590 | 3.208 | 2.210 |
| *NCOA3* | 1.475 | 5.479 | 2.700 | 3.146 | 2.440 |
| *DNAH1* | 1.457 | 5.753 | 2.340 | 2.932 | 2.160 |
| *ZMYM5* | 1.456 | 5.554 | 2.610 | 3.031 | 2.270 |
| *SP110* | 1.455 | 4.950 | 2.670 | 3.341 | 2.440 |
| *SGK223* | 1.437 | 5.478 | 2.950 | 2.994 | 2.320 |
| *PTPN6* | 1.422 | 5.397 | 2.420 | 2.974 | 2.200 |
| *PRPF38B* | 1.414 | 4.740 | 2.370 | 3.280 | 2.580 |
| *TEP1* | 1.413 | 5.525 | 2.400 | 2.871 | 2.050 |
| *ERVK13-1* | 1.409 | 4.709 | 2.010 | 3.277 | 2.430 |
| *TUBGCP6* | 1.396 | 6.198 | 2.590 | 2.456 | 1.900 |
| *SIGLEC10* | 1.392 | 5.471 | 3.310 | 2.816 | 2.220 |
| *TBC1D5* | 1.362 | 6.230 | 2.060 | 2.304 | 1.830 |
| *ADNP2* | 1.354 | 6.972 | 1.970 | 1.889 | 1.560 |
| *SP140* | 1.341 | 5.983 | 4.360 | 2.347 | 1.950 |
| *DNAJB14* | 1.337 | 5.259 | 2.320 | 2.701 | 2.070 |
| *TMEM260* | 1.331 | 6.505 | 2.050 | 2.034 | 1.670 |
| ***ERCC5*** | 1.320 | 6.148 | 2.420 | 2.175 | 1.680 |
| ***USP24*** | 1.310 | 5.103 | 1.990 | 2.674 | 1.980 |
| *ZNF700* | 1.307 | 6.165 | 2.490 | 2.114 | 1.700 |
| *ZNF430* | 1.304 | 6.628 | 2.400 | 1.862 | 1.600 |
| *PPM1K* | 1.302 | 5.240 | 3.160 | 2.571 | 2.050 |
| *NFX1* | 1.300 | 6.102 | 2.310 | 2.118 | 1.720 |
| ***EME2*** | 1.299 | 5.917 | 1.970 | 2.210 | 1.660 |
| *TRIM38* | 1.289 | 4.795 | 2.310 | 2.748 | 1.950 |
| *CA5BP1* | 1.277 | 5.020 | 2.410 | 2.582 | 2.000 |
| *MYCBP2* | 1.245 | 4.795 | 2.060 | 2.571 | 1.860 |
| *KPNA5* | 1.243 | 4.738 | 2.410 | 2.589 | 1.950 |
| *CEP350* | 1.242 | 5.472 | 1.950 | 2.207 | 1.760 |
| *PDCD7* | 1.227 | 5.130 | 2.030 | 2.324 | 1.750 |
| *FAM117B* | 1.224 | 5.063 | 2.760 | 2.347 | 2.010 |
| *AGO3* | 1.223 | 5.327 | 2.130 | 2.204 | 1.750 |
| *TTBK2* | 1.222 | 5.021 | 1.790 | 2.359 | 1.820 |
| *ZNF33A* | 1.213 | 5.609 | 2.500 | 2.018 | 1.710 |
| *PPP3CC* | 1.210 | 5.475 | 2.640 | 2.076 | 1.700 |
| *ZNF91* | 1.206 | 4.753 | 2.280 | 2.431 | 2.090 |
| *EDEM3* | 1.201 | 5.362 | 1.780 | 2.100 | 1.700 |
| *CD80* | 1.201 | 5.324 | 3.010 | 2.116 | 1.830 |
| *ZFP90* | 1.200 | 5.131 | 2.490 | 2.212 | 1.810 |
| *CELF2* | 1.196 | 4.932 | 2.090 | 2.299 | 1.860 |
| *METTL14* | 1.191 | 5.087 | 1.840 | 2.199 | 1.680 |
| *ATXN7* | 1.185 | 4.719 | 1.810 | 2.365 | 1.850 |
| *ARHGAP25* | 1.181 | 5.320 | 2.530 | 2.038 | 1.670 |
| *HPS5* | 1.165 | 5.290 | 2.530 | 1.990 | 1.650 |
| *HEATR5B* | 1.158 | 4.602 | 1.890 | 2.318 | 1.750 |
| *DMXL1* | 1.153 | 5.474 | 2.870 | 1.848 | 1.620 |
| *KMT2E* | 1.140 | 4.665 | 2.120 | 2.212 | 1.730 |
| *TTC13* | 1.138 | 4.529 | 1.860 | 2.274 | 1.730 |
| *MAST3* | 1.137 | 4.895 | 2.020 | 2.081 | 1.650 |
| *KIAA0430* | 1.129 | 4.493 | 1.960 | 2.254 | 1.830 |
| *MICAL1* | 1.123 | 4.786 | 2.350 | 2.080 | 1.700 |
| *C12orf66* | 1.113 | 4.840 | 2.450 | 2.010 | 1.660 |
| *THUMPD1* | 1.110 | 5.189 | 1.920 | 1.818 | 1.580 |
| *NAA35* | 1.109 | 4.518 | 1.710 | 2.161 | 1.780 |
| *SP100* | 1.096 | 4.942 | 2.280 | 1.891 | 1.590 |
| *RBBP6* | 1.094 | 4.649 | 2.070 | 2.031 | 1.660 |
| *SAMD9* | 1.092 | 4.629 | 2.030 | 2.033 | 1.690 |
| *DDX51* | 1.089 | 5.127 | 1.860 | 1.766 | 1.510 |
| *YTHDC2* | 1.087 | 4.777 | 2.060 | 1.940 | 1.630 |
| *WDFY4* | 1.071 | 4.804 | 2.430 | 1.861 | 1.600 |
| *DUS2* | 1.071 | 4.713 | 2.080 | 1.907 | 1.550 |
| *ACAA1* | 1.070 | 4.689 | 1.590 | 1.915 | 1.570 |
| *CHD9* | 1.062 | 4.497 | 1.930 | 1.981 | 1.650 |
| *NME6* | 1.030 | 4.638 | 2.070 | 1.780 | 1.550 |

**Supplementary Table 2B. SSGES genes uniquely under-expressed in SMZL.**

| **Gene** | **Impact Score** | **CSP d(i)** | **vCSP Fold-change** | **TBCL d(i)** | **vTBCL Fold-change** |
| --- | --- | --- | --- | --- | --- |
| *MEDAG* | 1.819 | -4.962 | -1.961 | -3.149 | -2.174 |
| *EGFR* | 1.785 | -5.082 | -3.125 | -2.969 | -2.439 |
| *SPINK2* | 1.689 | -3.994 | -1.563 | -3.270 | -3.226 |
| *CLEC1A* | 1.587 | -5.192 | -2.273 | -2.258 | -1.724 |
| *TCTN2* | 1.496 | -4.831 | -1.923 | -2.167 | -1.754 |
| *LOC101929398* | 1.474 | -5.794 | -1.818 | -1.549 | -1.429 |
| *PLS1* | 1.460 | -4.905 | -2.381 | -2.006 | -1.695 |
| *PALD1* | 1.426 | -4.259 | -1.639 | -2.260 | -1.818 |
| *CDK15* | 1.414 | -4.854 | -1.818 | -1.883 | -1.538 |
| *RARB* | 1.406 | -5.135 | -1.961 | -1.700 | -1.493 |
| *HS3ST2* | 1.384 | -4.233 | -1.587 | -2.136 | -1.695 |
| *NPAS2* | 1.378 | -5.671 | -2.222 | -1.307 | -1.351 |
| *CBX2* | 1.347 | -4.405 | -1.923 | -1.920 | -1.587 |
| *PTGFRN* | 1.343 | -5.079 | -2.083 | -1.525 | -1.429 |
| *SLC16A2* | 1.320 | -5.014 | -1.667 | -1.485 | -1.408 |
| *CDH6* | 1.303 | -4.795 | -2.128 | -1.555 | -1.449 |
| *CSMD2-AS1* | 1.298 | -4.978 | -1.887 | -1.435 | -1.389 |
| *FMN1* | 1.281 | -4.708 | -1.613 | -1.532 | -1.408 |
| *NRIP3* | 1.279 | -5.119 | -1.724 | -1.294 | -1.351 |
| *LOC105374690* | 1.248 | -4.750 | -1.695 | -1.401 | -1.370 |
| *ADCYAP1R1* | 1.244 | -4.854 | -1.613 | -1.327 | -1.333 |
| *CD177* | 1.241 | -4.958 | -1.818 | -1.260 | -1.316 |
| *NMNAT2* | 1.232 | -3.887 | -1.538 | -1.834 | -1.493 |
| *LOC101928435* | 1.230 | -4.624 | -1.695 | -1.412 | -1.370 |
| *MTA2* | 1.228 | -4.127 | -1.538 | -1.688 | -1.493 |
| *OR7E12P* | 1.226 | -4.066 | -1.587 | -1.713 | -1.471 |
| *HOXD1* | 1.208 | -4.629 | -1.613 | -1.339 | -1.351 |
| *ELK2AP* | 1.205 | -4.194 | -1.515 | -1.572 | -1.408 |
| *LOC100652911* | 1.203 | -4.454 | -1.667 | -1.421 | -1.389 |
| *BTNL3* | 1.197 | -4.760 | -1.667 | -1.227 | -1.299 |
| *OR10A5* | 1.182 | -4.273 | -1.667 | -1.453 | -1.389 |
| *LOC646482* | 1.179 | -4.100 | -1.667 | -1.543 | -1.449 |
| *LOC101928132* | 1.162 | -4.346 | -1.667 | -1.346 | -1.351 |
| *OR1J2* | 1.159 | -4.138 | -1.695 | -1.454 | -1.389 |
| *SYT2* | 1.159 | -3.938 | -1.538 | -1.566 | -1.449 |
| *CD248* | 1.155 | -3.836 | -1.429 | -1.613 | -1.471 |
| *LOC339666* | 1.149 | -4.439 | -1.563 | -1.253 | -1.333 |
| *DCUN1D3* | 1.141 | -4.461 | -1.471 | -1.215 | -1.299 |
| *AGPAT1* | 1.137 | -4.151 | -1.515 | -1.375 | -1.370 |
| *MDH1B* | 1.123 | -4.085 | -1.639 | -1.368 | -1.351 |
| *PRSS33* | 1.122 | -4.418 | -1.613 | -1.176 | -1.299 |
| *CORT* | 1.119 | -3.740 | -1.471 | -1.549 | -1.408 |
| *MAS1* | 1.111 | -4.002 | -1.515 | -1.375 | -1.351 |
| *RTP3* | 1.109 | -4.054 | -1.587 | -1.340 | -1.351 |
| *ELK1* | 1.106 | -4.093 | -1.493 | -1.307 | -1.316 |
| *SLC6A13* | 1.096 | -3.934 | -1.471 | 2.062 | -0.595 |
| *HOXC11* | 1.087 | -3.896 | -1.563 | -1.356 | -1.351 |
| *LOC105376292* | 1.087 | -4.240 | -1.538 | -1.160 | -1.282 |
| *LOC100131303* | 1.085 | -3.802 | -1.493 | -1.402 | -1.370 |
| *MKS1* | 1.068 | -4.166 | -1.587 | -1.141 | -1.282 |
| *TRPV3* | 1.061 | -3.935 | -1.493 | -1.250 | -1.316 |
| *SLC18A2* | 1.061 | -4.097 | -1.471 | -1.157 | -1.282 |
| *ASAP3* | 1.056 | -3.749 | -1.493 | -1.338 | -1.351 |
| *KRBA1* | 1.045 | -4.000 | -1.471 | -1.159 | -1.282 |
| *SLC17A3* | 1.039 | -3.889 | -1.493 | -1.204 | -1.316 |
| *OR4D2* | 1.038 | -3.781 | -1.493 | -1.261 | -1.316 |
| *SCN3B* | 1.023 | -3.872 | -1.471 | -1.159 | -1.282 |
| *ETNK2* | 1.020 | -3.898 | -1.493 | -1.136 | -1.282 |
| *LOC100506047* | 0.998 | -3.752 | -1.449 | -1.147 | -1.282 |
| *CYP4B1* | 0.993 | -3.754 | -1.408 | -1.129 | -1.282 |
| *GABRQ* | 0.974 | -3.481 | -1.389 | -1.219 | -1.316 |
| *CDRT1* | 0.957 | -3.738 | -1.449 | -1.020 | -1.250 |
| *LAMA3* | 0.908 | -3.244 | -1.333 | -1.137 | -1.282 |
