# Supplementary material for: Identification of a Splenic Marginal Zone Lymphoma Signature: Preliminary Findings With Diagnostic Potential
Source: Front Oncol. 2020 May 8;10:640. doi: 10.3389/fonc.2020.00640 (PMC7225304; doi:10.3389/fonc.2020.00640)
Supplement: Supplementary file 3 [file Table_3.docx]

**Supplementary Table 3. Gene Expression Validation Datasets.**

All datasets utilized in the validation cohort of this study are listed. The reference dataset ID for GEO, the amount and subtype of samples included, and the PubMed ID for publications associated with the data are provided.

| **GEO Dataset ID** | **Sample Type (n)** | **Associated Reference PMID** |
| --- | --- | --- |
| GSE2109 | Control Spleen (3), SMZL (1) | N/A |
| GSE55267 | Follicular Lymphoma (63), B-cell control (6) | 24634383 |
| GSE57520 | Hepatosplenic T-cell Lymphoma (4), Control Spleen (3) | 25057852 |
| GSE7307 | Control Spleen (5) | N/A |
| GSE70910 | Mantle Cell Lymphoma (34) | 27127301 |
| GSE7788 | Nodular Lymphocyte Predominant Hodgkin's Lymphoma (10), T-cell/Histocyte Rich B cell Lymphoma (10) | 19797726 |
| GSE74266 | Diffuse Large B-cell Lymphoma (28) | 26854484 |
| GSE86613 | Follicular Lymphoma (7), Transformed Follicular Lymphoma (2), Diffuse Large B-cell Lymphoma (41) | 28097046 |
| GSE93261 | Follicular Lymphoma (149) | 29475724 |
| GSE93984 | Diffuse Large B-cell Lymphoma (88) | 28428442 |
| GSE16455 | SMZL (4) [PB] | 20124476 |
| GSE17372 | HIV+ Burkitt Lymphoma (4), HIV+ Diffuse Large B-cell Lymphoma (13) | 20216076 |
| GSE17920 | Hodgkin Lymphoma (130) | 20220182 |
| GSE25550 | Control Spleen (6), Extranodal Marginal Zone Lymphoma (14) | 21273489, 22058210 |
| GSE25638 | SMZL [PB] (12) | 22689981 |
| GSE26673 | HIV+ Burkitt Lymphoma (2), Burkitt Lymphoma (14) | 21245480 |
| GSE35082 | SMZL [PB] (9) | 22816737 |
| GSE35348 | SMZL (27) | N/A |
| GSE35426 | SMZL (14) | 23028731 |
| GSE2109 | Control Spleen (3), SMZL (1) | N/A |
| GSE55267 | Follicular Lymphoma (63), B-cell control (6) | 24634383 |
| GSE57520 | Hepatosplenic T-cell Lymphoma (4), Control Spleen (3) | 25057852 |
| GSE146814 | SMZL (1) | N/A |
|  | Total: 705 |  |
