# Supplementary material for: Identification of a Splenic Marginal Zone Lymphoma Signature: Preliminary Findings With Diagnostic Potential
Source: Front Oncol. 2020 May 8;10:640. doi: 10.3389/fonc.2020.00640 (PMC7225304; doi:10.3389/fonc.2020.00640)
Supplement: Supplementary file 4 [file Table_4.docx]

**Supplementary Table 4. Patient Prognostic Information.**

Each splenic sample utilized for IHC was previously diagnosed by a trained pathologist. All samples were acquired from the Nebraska Lymphoma Study Group Tissue Bank at UNMC, and this study was reviewed and approved by the Institutional Review Board at UNMC, #441-18-EP.

| ***SMZL*** | *diagnosis* | *stage* | *symptom status* |
| --- | --- | --- | --- |
| SMZL1 | SMZL | 3 | A |
| SMZL2 | SMZL | 4 | B |
| SMZL3 | SMZL | 4 | A |
| SMZL4 | SMZL | NA | NA |
| SMZL5 | SMZL | NA | NA |
| SMZL6 | SMZL | 4 | B |
|  |  |  |  |
| ***CSP*** | *diagnosis* | *stage* | *symptom status* |
| CSP1 | NEG | 3 | A |
| CSP2 | NEG | 2 | A |
| CSP3 | NEG | 3 | A |
| CSP4 | NEG | 2 | B |
| CSP5 | NEG | 3 | A |
| CSP6 | NEG | NA | NA |
|  |  |  |  |
| ***TBCL*** | *diagnosis* | *stage* | *symptom status* |
| DLCL1 | DLCL-C | NA | NA |
| DLCL2 | DLCL-NC | 2 | A |
| FL1 | FL-3 | NA | NA |
| FM1 | FM | 4 | A |
| FM2 | FM | 4 | A |
