# Supplementary material for: Identification of a Splenic Marginal Zone Lymphoma Signature: Preliminary Findings With Diagnostic Potential
Source: Front Oncol. 2020 May 8;10:640. doi: 10.3389/fonc.2020.00640 (PMC7225304; doi:10.3389/fonc.2020.00640)
Supplement: Supplementary file 5 [file Table_5.docx]

**Supplementary Table 5. Immunohistochemistry Antibody Information.**

Antibody information and optimization description utilized for immunohistochemistry staining of 5 micron splenic slides. Immunohistochemistry was executed using *Roche Diagnostics Discovery* platform at the University of Nebraska Medical Center.

| **Antibody** | **Company** | **Catalog No.** | **Antigen Retrieval Time (min)** | **Antibody Dilution** | **Antibody Primary Stain Time (min)** |
| --- | --- | --- | --- | --- | --- |
| EME2 | Biorbyt | orb357635 | 24 | 1:1000 | 48 |
| ZBTB32 | Biorbyt | orb183380 | 32 | 1:200 | 48 |
| ERCC5 | Bethyl Lab. Inc. | IHC-00529 | 32 | 1:150 | 48 |
| USP24 | Sigma-Aldrich | SAB4500975 | 32 | 1:25 | 60 |
| SETBP1 | Invitrogen | PA5-61863 | 32 | 1:50 | 48 |
