# Supplementary material for: Identification of a Splenic Marginal Zone Lymphoma Signature: Preliminary Findings With Diagnostic Potential
Source: Front Oncol. 2020 May 8;10:640. doi: 10.3389/fonc.2020.00640 (PMC7225304; doi:10.3389/fonc.2020.00640)
Supplement: Supplementary file 6 [file Table_6.docx]

**Supplementary Table 6. Multiple method predictive analysis of SSGES for identifying SMZL.**

The table is separated into six sub-tables (A. – F.). **A.** Multiple method predictive analysis for SMZL compared with CSP using the SSGES. **B.** Multiple method predictive analysis for SMZL compared with TBCLs using the SSGES. **C.** Multiple method predictive analysis for SMZL compared with CSP using the 5 gene IHC panel. **D.** Multiple method predictive analysis for SMZL compared with TBCLs using the 5 gene IHC panel. **E.** Multiple method predictive analysis for SMZL compared with CSP using the 5 gene IHC panel in the replication sample cohort. **F.** Multiple method predictive analysis for SMZL compared with TBCLs using the 5 gene IHC panel in the replication sample cohort. All tables list the 7 methodologies used for prediction, as well as the average percentage that samples were correctly classified based on the signature.

**Supplementary Table 6A. Predicted SMZL identification when compared with CSP using the SSGES.**

| **Predictive Analysis Method** | **Mean percent of correct classification:** |
| --- | --- |
| Compound Covariate Predictor | 100 |
| Diagonal Linear Discriminant Analysis | 100 |
| 1-Nearest Neighbor | 100 |
| 3-Nearest Neighbors | 100 |
| Nearest Centroid Correct | 100 |
| Support Vector Machines | 100 |
| Bayesian Compound Covariate Predictor | 100 |

**Supplementary Table 6B. Predicted SMZL identification when compared with TBCLs using the SSGES.**

| **Predictive Analysis Method** | **Mean percent of correct classification:** |
| --- | --- |
| Compound Covariate Predictor | 96 |
| Diagonal Linear Discriminant Analysis | 96 |
| 1-Nearest Neighbor | 99 |
| 3-Nearest Neighbors | 100 |
| Nearest Centroid Correct | 97 |
| Support Vector Machines | 99 |
| Bayesian Compound Covariate Predictor | 100 |

**Supplementary Table 6C. Predicted SMZL identification when compared with CSP using the 5-gene IHC Panel.** *(ERCC5, EME2, SETBP1, USP24, ZBTB32)*

| **Predictive Analysis Method** | **Mean percent of correct classification:** |
| --- | --- |
| Compound Covariate Predictor | 98 |
| Diagonal Linear Discriminant Analysis | 98 |
| 1-Nearest Neighbor | 98 |
| 3-Nearest Neighbors | 98 |
| Nearest Centroid Correct | 98 |
| Support Vector Machines | 98 |
| Bayesian Compound Covariate Predictor | 98 |

**Supplementary Table 6D. Predicted SMZL identification when compared with TBCLs using the 5-gene IHC Panel.** *(ERCC5, EME2, SETBP1, USP24, ZBTB32)*

| **Predictive Analysis Method** | **Mean percent of correct classification:** |
| --- | --- |
| Compound Covariate Predictor | 92 |
| Diagonal Linear Discriminant Analysis | 91 |
| 1-Nearest Neighbor | 93 |
| 3-Nearest Neighbors | 95 |
| Nearest Centroid Correct | 91 |
| Support Vector Machines | 97 |
| Bayesian Compound Covariate Predictor | 96 |

**Supplementary Table 6E. Predicted SMZL identification against the replication cohort when compared with CSP using the 5-gene IHC Panel.** *(ERCC5, EME2, SETBP1, USP24, ZBTB32)*

| **Predictive Analysis Method** | **Mean percent of correct classification:** |
| --- | --- |
| Compound Covariate Predictor | 98 |
| Diagonal Linear Discriminant Analysis | 100 |
| 1-Nearest Neighbor | 97 |
| 3-Nearest Neighbors | 100 |
| Nearest Centroid Correct | 95 |
| Support Vector Machines | 98 |
| Bayesian Compound Covariate Predictor | 98 |

**Supplementary Table 6F. Predicted SMZL identification against the replication cohort when compared with TBCL using the 5-gene IHC Panel.** *(ERCC5, EME2, SETBP1, USP24, ZBTB32)*

| **Predictive Analysis Method** | **Mean percent of correct classification:** |
| --- | --- |
| Compound Covariate Predictor | 92 |
| Diagonal Linear Discriminant Analysis | 90 |
| 1-Nearest Neighbor | 98 |
| 3-Nearest Neighbors | 97 |
| Nearest Centroid Correct | 89 |
| Support Vector Machines | 97 |
| Bayesian Compound Covariate Predictor | 97 |
