# Supplementary material for: Identification of a Splenic Marginal Zone Lymphoma Signature: Preliminary Findings With Diagnostic Potential
Source: Front Oncol. 2020 May 8;10:640. doi: 10.3389/fonc.2020.00640 (PMC7225304; doi:10.3389/fonc.2020.00640)

Supplementary Figure 1. Immunophenotypic classification of SMZL.
Immunophenotyping of CSP samples as well as malignant SMZL cases using immunohistochemistry. (A-D) BCL2, positive in SMZL cells. (E-H) BCL6, negative in SMZL cells. (I-L) CD10, negative in SMZL cells.

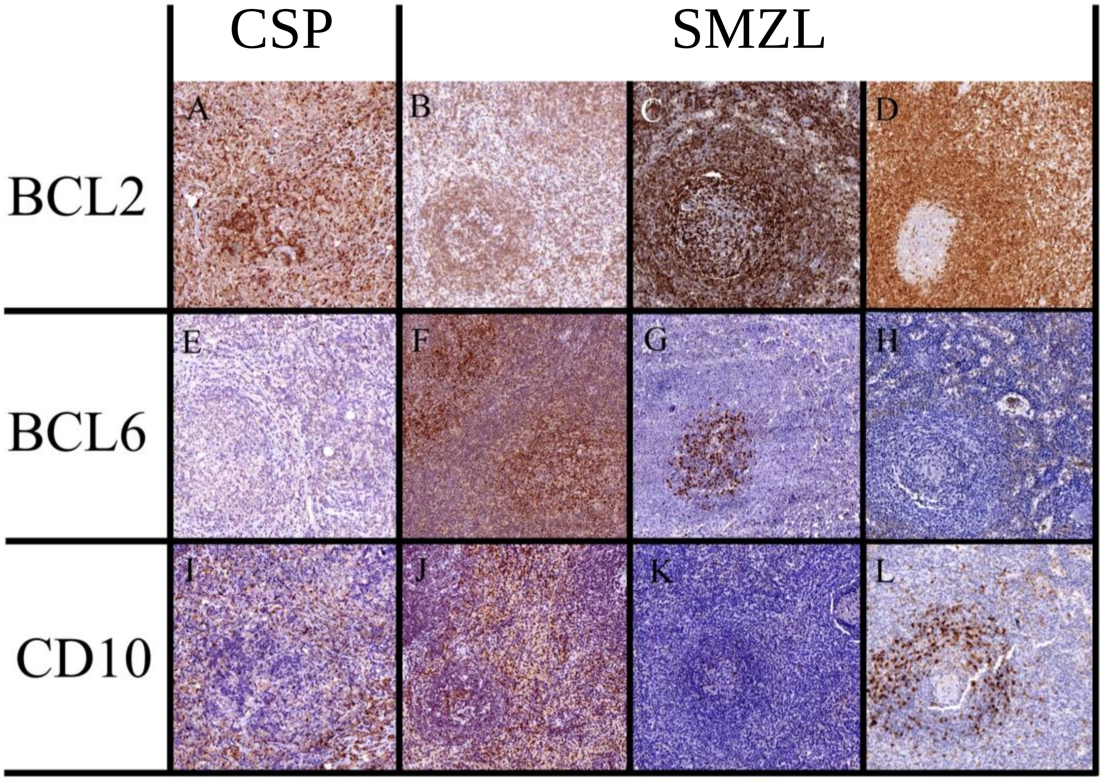

Supplement: Supplementary file 7 [file Image_1.PDF]
